# Supplementary material for: Inflammatory factors and risk of lung adenocarcinoma: a Mendelian randomization study mediated by blood metabolites
Source: Front Endocrinol (Lausanne). 2024 Aug 27;15:1446863. doi: 10.3389/fendo.2024.1446863 (PMC11384989; doi:10.3389/fendo.2024.1446863)
Supplement: Supplementary file 1 [file DataSheet1.zip › supplementary files/S2/GCST90200866/forest.pdf]

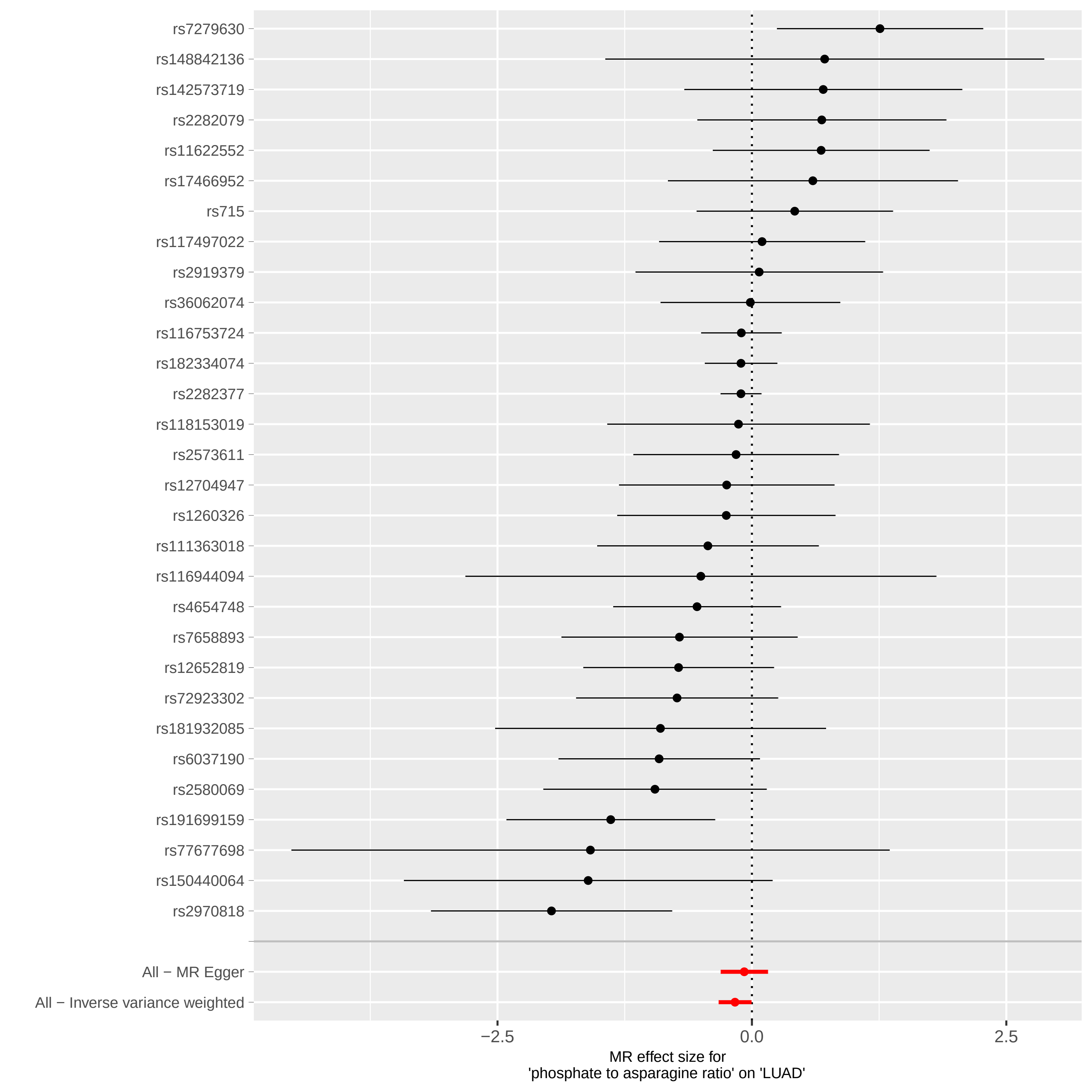
rs7279630
rs148842136
rs142573719
rs2282079
rs11622552
rs17466952
rs715
rs117497022
rs2919379
rs36062074
rs116753724
rs182334074
rs2282377
rs118153019
rs2573611
rs12704947
rs1260326
rs111363018
rs116944094
rs4654748
rs7658893
rs12652819
rs72923302
rs181932085
rs6037190
rs2580069
rs191699159
rs77677698
rs150440064
rs2970818
All − MR Egger
All − Inverse variance weighted
−2.5
0.0
2.5
MR effect size for
'phosphate to asparagine ratio' on 'LUAD'
